# Evaluation of biological synthesized platinum nanoparticles using *Ononidis radix* extract on the cell lung carcinoma A549

**DOI:** 10.1007/s10544-019-0424-7

**Published:** 2019-07-25

**Authors:** Renata Dobrucka, Aleksandra Romaniuk-Drapała, Mariusz Kaczmarek

**Affiliations:** 1 Department of Industrial Products Quality and Ecology, Faculty of Commodity Science, Poznan University of Economics, al. Niepodległości 10, 61-875 Poznan, Poland; 2 Department of Clinical Chemistry and Molecular Diagnostics, Poznan University of Medical Sciences, 49 Przybyszewskiego St, 60-355 Poznań, Poland; 3 Department of Immunology, Chair of Clinical Immunology, Poznan University of Medical Sciences, Rokietnicka 5D, 60-806 Poznan, Poland

**Keywords:** Platinum nanopartilces, Green synthesis, Anticancer activity

## Abstract

Due to the search for new methods for synthesizing nanomaterials, this work proposes the biological synthesis of platinum nanoparticles using *Ononidis radix* extract. The synthesized platinum nanoparticles were characterized by UV-Vis, Scanning Electron Microscopy (SEM) with EDS profile, Fourier transform infrared spectroscopy (FTIR), Transmission Electron Microscopy (TEM) and Atomic Force Microscopy (AFM). The examination conducted by means of Transmission Electron Microscopy showed the presence of spherical and hexagonal platinum nanoparticles. Atomic Force Microscopy indicated the presence of locally agglomerated nanoparticles whose size was about 4 nm. The study also examined the influence of platinum nanoparticles on human non-small cell lung carcinoma cells A549. It was found that the mortality of cells cultured together with platinum nanoparticles increased, and the proliferative activity of A549 cells decreased gradually over time in proportion to the increasing concentration of the test substance.

**Figure:**
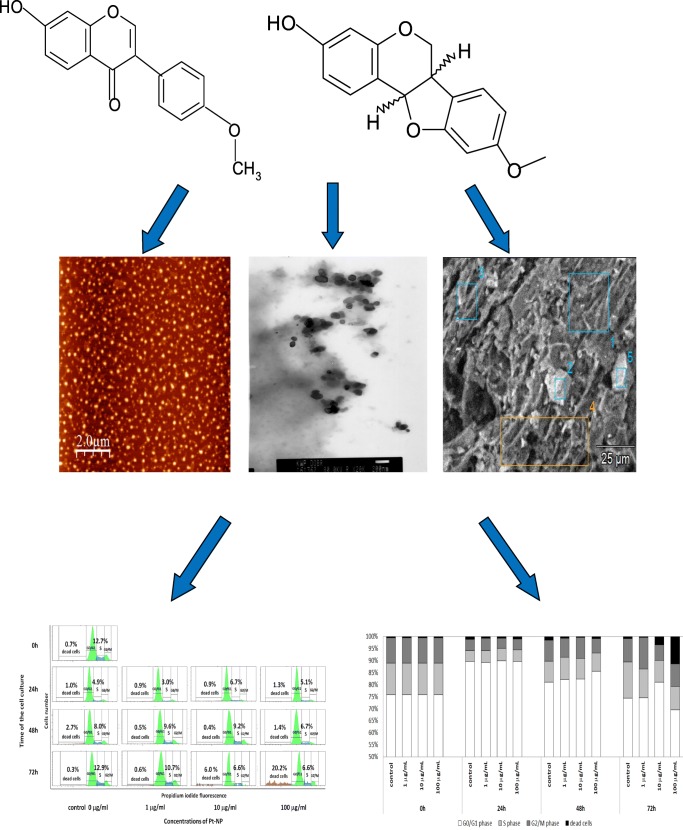
Graphical abstract

Graphical abstract

## Introduction

Nanoparticles are a group of materials with morphological properties smaller than a micrometer in at least one external dimension, or with a nanoscale internal structure. As a result of those changes, nanoparticles have a large surface area, which is chemically more reactive than their fine structural analogues (Ajitha et al. 2015; Bhatnagar et al. 2018). The most popular method for synthesizing nanoparticles is the chemical reduction method, which uses chemical substances as reducing agents. Even though chemical syntheses have several advantages, the use of toxic chemicals on the surface of nanoparticles and non-polar solvents in the synthesis procedure limits their applications in clinical fields (Vijayaraghavan and Ashokkumar 2017). Researchers have tried to develop clean, biocompatible, non-toxic and eco-friendly methods for synthesizing nanoparticles (Dauthal and Mukhopadhyay 2016). Such an alternative may be offered by biological synthesis, which is developing rapidly due to its growing success and ease of formation of nanoparticles.

At present, scientists are carrying out intense studies focused on synthesizing metal nanoparticles with the use of various microorganisms, such as bacteria, algae, actinomycetes, fungi, yeast and viruses (Narayanan and Sakthivel 2010; Thakkar et al. 2010). If algae or plants are used, the production of nanoparticles is usually limited to the extracellular method. The use of plant extracts in biological synthesis has certain advantages: they are easily available, safe to handle, and they possess a broad viability of metabolites. Moreover, it has been found that plant extracts act both as reducing and capping agents in the process of synthesizing nanoparticles (Nasrollahzadeh et al. 2016; Baranwal et al. 2018a, b). The reduced form of metals obtained from biological synthesis is highly reactive and used in various fields, like the biological field, electrochemistry and photochemistry (Khan et al. 2017). One of great advantages of biologically synthesized nanomaterials is that they have potential applications in different areas, such as medicine or pharmacy (Baranwal et al. 2016). Matal nanoparticles are used as antimicrobial agents (Baranwal et al. 2018a, b). The world literature contains numerous examples of the biological synthesis of metal nanoparticle, but biological methods for synthesizing platinum nanoparticles with the use of plant materials have not been widely exploited. The literature presents several examples of the green synthesis of platinum nanoparticles with the use of, for example, leaves of *Cerbera manghas* (Rajathi and Nambaru 2014), *Azadirachta indica* (Thirumurugan et al. 2016), quail egg yolk (Nadaroglu et al. 2017), *Fumariae herba* (Dobrucka 2016) or pomegranate extract (Şahin et al. 2018).

In this work, the synthesis of platinum nanoparticles was carried out with the use of *Ononis spinosa L*, which is a species belonging to the *Fabaceae* family. It grows in Europe and on the sandy areas of Asia. It has trifoliate leaves and papilionaceous flowers. The roots of *Ononis spinosa L* are ligneous and twisted, and due to their medicinal properties, they are often used in herbal medicine. *Ononidis radix* contains flavonoid derivatives, sterols, terpenic substances, phenolic acids and essential oil. Due to the high content of biologically active substances, *O.radix* was used in this study to carry out the biological synthesis of nanoparticles.

## Materials and methods

### Materials

All the reagents used in this study were purchased from Sigma–Aldrich (Poland). Milli-Q water was used throughout the experiment. All chemicals were of analytical grade.

### Synthesis of platinum nanoparticles

Healthy samples of *O.radix was obtained from* Wielkopolska region (Poland, 51°52′56″N, 17°00′44″E). To 3 g powdered of *O. radix*, there were added 100 mL of double distilled water. The solution was boiled and stirred for 1 h at the temperature of 85 °C. Next, 0.02 g K_2_PtCl_6_ was added into the cooled 30 mL extract of *O. radix.* This solution was stirred for 5 h in temperature 80 °C. After 10 h, the UV-absorption spectrum of the synthesized platinum nanoparticles was monitored.

### Characterization of platinum nanoparticles

The analysis of optical property of biological synthesized platinum nanoparticles using *O. radix* s was made using ultraviolet and visible absorption spectroscopy (spectrophotometer Cary E 500) in the range of 250 nm–600 nm. In order to characterize the synthesized platium nanoparticles, there was conducted Fourier transform infrared spectroscopy (FTIR) using Perkin Elmer Spectrum 1000, in attenuated total reflection mode and using spectral range of 4000–380 cm − 1, with a resolution of 4 cm − 1. The structure of biological synthesized platinum nanoparticles was studied using TEM (Transmission electron microscope) JEOL JEM 1200 EXII, operating at 80 kV. In this work AFM (atomic force microscope) INTEGRA SPECTRA SOLAR of NT-MDT brand and measurement tips dedicated for NSGO1 high-resolution measurements was used. The **picture of** biological synthesized platinum nanoparticles was prepared by means of SEM (scanning electron microscopy) SEM **SU3500, Hitachi with spectral imaging system Thermo Scientific NSS (EDS).**

### Evaluation of the cell cycle

The influence of platinum nanoparticles on human cells was evaluated *in vitro* using the established epithelial cell line of human non-small cell lung carcinoma A549 from American Type Culture Collection (ATCC® CCL-185™). Cells were cultured in Nunc Easy Flask 75cm^2^ (Thermo Fisher Scientific) in RPMI-1640 culture medium with 2 mM L-glutamine (LONZA) supplemented with 10% fetal bovine serum (FBS; Sigma-Aldrich) and with 1% of a solution of antibiotic-antimycotic mixture contained 10000 U/mL penicillin, 10 mg/mL streptomycin and 25 μl/mL amphotericin B (Sigma-Aldrich). The cultures were kept at 37 °C in an incubator in a humidified 5% CO_2_ atmosphere. Cells prepared for the test were transferred in suspension 4 × 10^4^ cells per well to 24-well flat-bottomed plastic plates (TC-PLATE 24 wells, Greiner) and incubated for 24 h to achieve adhesion. In the course of the test, cells were treated with platinum nanoparticles in followed concentrations: 1 μg/mL, 10 μg/mL and 100 μg/mL, and then were cultured for 24, 48, and 72 h. As a control, the cells cultured without tested platinum nanoparticles solutions were used. All tests were made in triplicates.

**Fig. 1:**
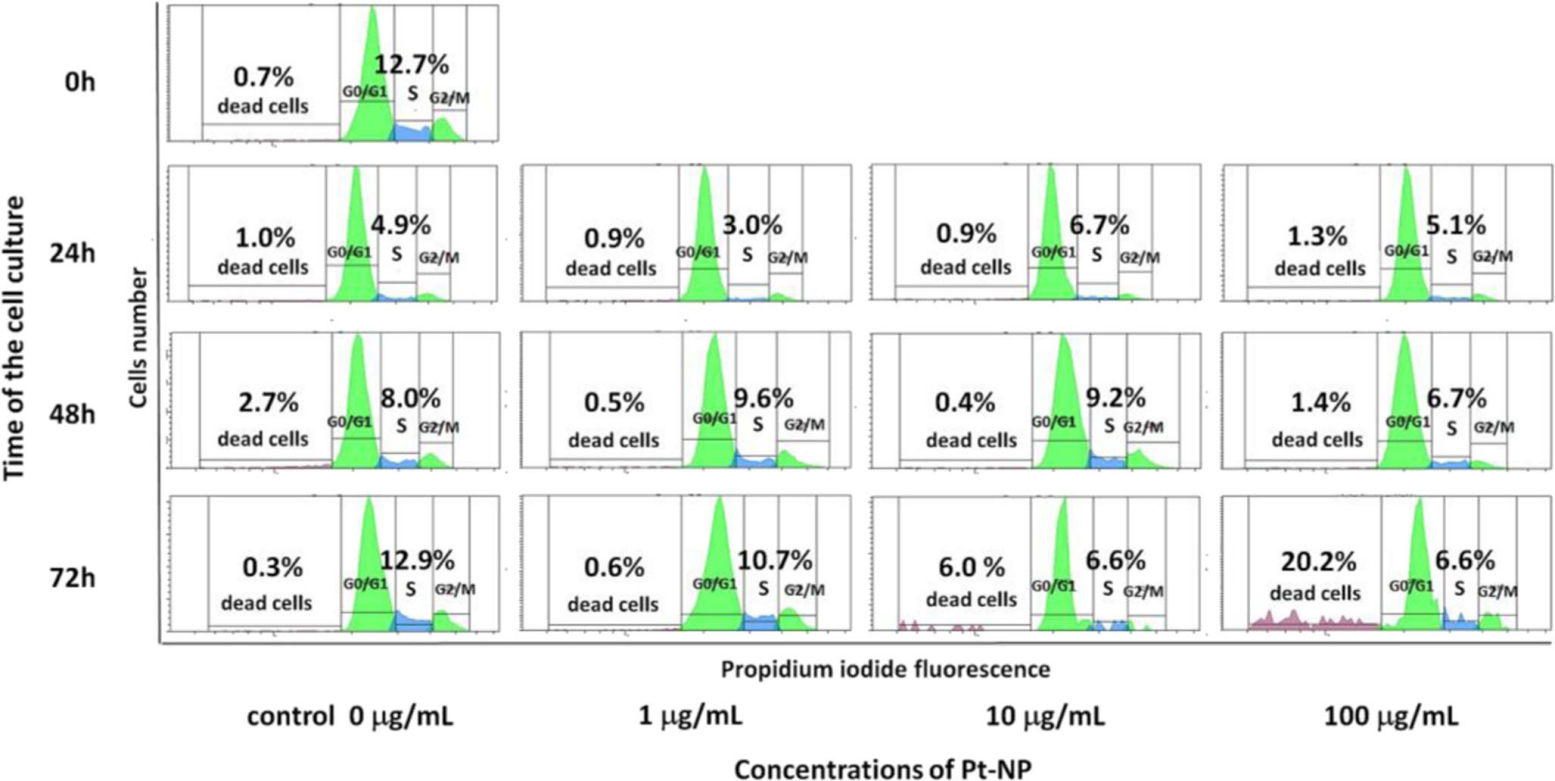
Histograms presented the cell cycle analysis of the A549 cell line cultured under the influence of platinum nanoparticles in 1 μg/mL, 10 μg/mL, and 100 μg/mL concentration

The platinum nanoparticles effect on A549 cells was investigated by the cell cycle evaluation. During the test, the cells stained with propidium iodide, the fluorochrome substance which intercalates into the DNA of cells, were analyzed by use flow cytometer. This approach allows for measurement of the percentage of cells in each cell cycle phases as well as the proportion of dead cells in the studied sample (Fig. 1). On the basis of the percentage of cells in S phase, we estimated the proliferative activity of cells, also cells before mitosis in the G2/M phase. After incubation with platinum nanoparticles, the cells were detached from plates by use 0,25% Trypsin-EDTA buffer (Biowest) and washed twice with PBS buffer. Next, cell pellets were re-suspended in 500 μl of cold permeabilization buffer, BD Perm/Wash Buffer (BD Biosciences), and incubated for 30 min at 4 °C. After centrifugation at 400 g for 5 min, pellets were re-suspended in 1 mL cold PBS containing 10 mg/mL propidium iodide (Sigma-Aldrich) and 100 U/mL RNase enzyme (Boehringer Mannheim), and incubated for 30 min at 4 °C protected from light. Then, the samples were acquired by using the flow cytometer FACS Canto (Becton Dickinson) and analyzed using FACS Diva software (Becton Dickinson). The results were tested with one-way non-parametric ANOVA (Kruskal-Wallis test).

## Results and discussion

### UV VIS studies of platinum nanoparticles

**Fig. 2:**
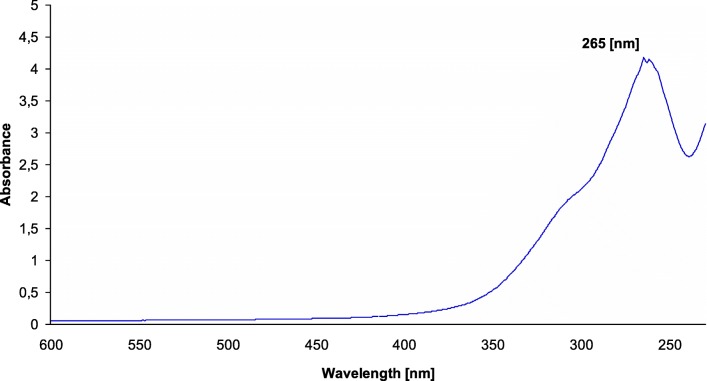
UV–vis absorption spectrum of synthesized platinum nanopartilces using *O.radix* extract

UV–vis spectroscopy is absorption spectroscopy used to confirm the synthesis of nanoparticles and nanocomposites. It was employed in this work to determine the presence of synthesized platinum nanoparticles. Figure 2 presents the UV–visible spectra of platinum nanoparticles synthesized using *O.radix* extract. The absorbance was measured after the solution had been stirred magnetically for 5 h at the temperature of 80 °C, and the absorption peak was recorded at 265 nm. The reaction was continued for the next 5 h. After that time, it was observed that as the incubation time increased, the intensity of the absorption peak at 265 nm significantly lowered. Finally, it disappeared and was replaced by a broad continuous absorption spectrum between 200 and 300 nm, which indicated the formation of platinum nanoparticles (Leo and Oluwafemi 2017). During mixing, a change of color was observed: from yellow to yellowish brown. This indicated that platinum nanoparticles were formed due to the reduction process of Pt^4+^ to Pt^0^ nanoparticles.

### Fourier transform infrared spectroscopy (FTIR) studies of platinum nanoparticles

**Fig. 3:**
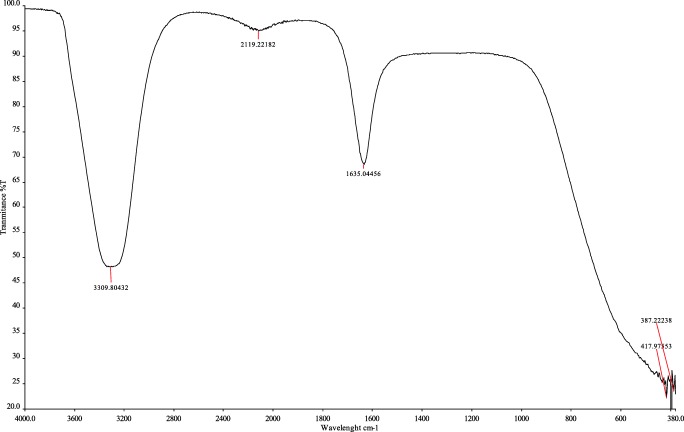
FT-IR spectra of synthesized platinum nanopartilces using *O. radix* extract

**Fig. 4:**
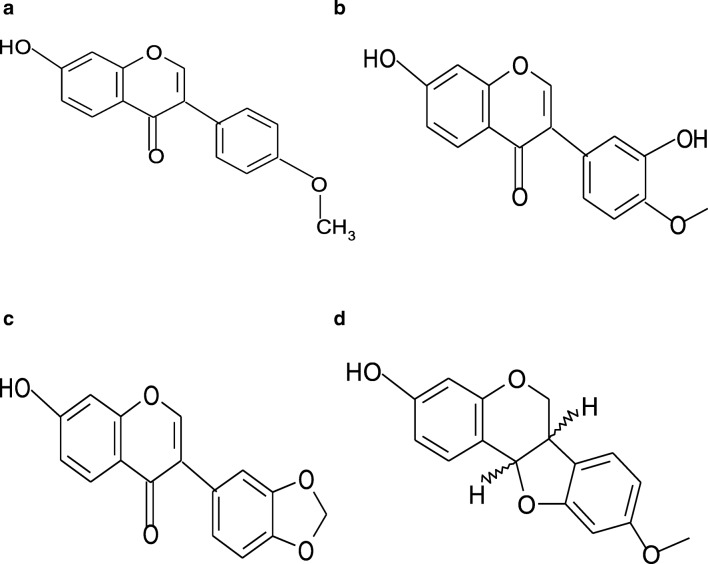
The chemical structure of of isoflavonoids: **a** formononetin, **b** calycosin, **c** pseudobaptigenin and (**d**) medicarpin

Figure 3 presents the results of the FTIR analysis of platinum nanoparticles synthesized using *O. radix* extract. To find the functional groups involved in the bioreduction of platinum nanoparticles, Fourier transform infrared spectroscopy (FTIR) measurements were carried out. The strong peaks were observed at 3309 cm − 1, 2119 cm − 1, 1635 cm − 1, 417 cm − 1 and 387 cm−. The strong absorption peak that appeared at 3309 cm-1 is related to O-H stretching (intramolecular hydrogen bonded OH). The band which appeared at 2119 cm-1 indicates the presence of an alkyne group. The most intense band at 1635 cm − 1 represents vibrations C=O, typical of the structure of flavonoids. The most intense band at 387 cm-1 is attributed to the deformation vibration of C­C in polymer chains. Fourier Transform Infrared Spectroscopy (FTIR) also confirmed that *O.radix* contained bioactive compounds, which served as reducing and capping agents for Pt nanoparticles. The phytochemical studies on *O. radix* have determined the presence of flavonoid derivatives, sterols (β-sitosterol, campesterol, stigmasterol, stigmastan-3,5-dien), terpenic substances, phenolic acids and essential oil (carvone, menthol, menthone, isomenthone, linalool, estragole, borneol and cis-anethole (Öz et al. 2018). Another significant group of biologically active compounds found in *O. radix* are isoflavonoids. Isoflavonoids have a phenyl substituent at the 3-position of the chromane ring (3-phenylchromen-2-one-4 or 3-phenylchromen--4-one). In the studies conducted by Gampe et al. (2016), isoflavonoids (formononetin, calycosin and pseudobaptigenin), pterocarpans (medicarpin and maackiain) and dihydroisoflavonoids (onogenin and sativanone) were found in the forms of glucosides, glucoside malonates, glucoside acetates and aglycones. The studies carried out by Gampe et al. in 2018 additionally confirmed the presence of six piperidin-2-ylacetic acid (homopipecolic acid) esters of isoflavonoid glucosides. Isoflavonoids are responsible for various biological activities, such as estrogenic, antioxidant and anticancer activities (Sharma and Ramawat 2013). In the studies conducted by Wang et al. (2013) the aglycone of sativanone-7-O-glucoside was shown to possess anti-inflammatory properties *in vitro*. Moreover, it was found that sativanone inhibited the release of TNF-α from polysaccharide activated macrophages. Sativanone was also found to induce expression of collagen type I and transforming growth factor-b1 in human dermal fibroblasts (Ham et al. 2015). According to the literature (Wilska-Jeszka and Podsędek 2001), the antioxidant properties of flavonoids are determined by the presence of hydroxy groups in both rings, the isomerism of their position, and the presence of a double bond and a carbonyl group in the heterocyclic ring. A stronger antioxidant potential is exhibited by flavonoids which have a greater number of hydroxy groups that are located in the para position. In addition, it was discovered that the differences in antioxidant properties also depend on the type of the saccharide residue present in the particle. *O. radix* also contains phenolic acids (p-hydroxybenzoic, vanillic acid, caffeic acid, syringic acid, p-coumaric acid, cinnamic acid, sinapin acid, salicylic acid, gentisin acid), which have antioxidant properties as well. Sinapinic acid with two methoxy groups is more active than ferulic acid, which has one methoxy group, and the latter is more active than coumaric acid (which has one hydroxy group) (Rice-Evans et al. 1996). Figure 4 presents the chemical structure of isoflavonoids: (A) formononetin, (B) calycosin, (C) pseudobaptigenin and (D) medicarpin.

### Transmission (TEM) and scanning Electron microscopy (SEM) with EDS profile of platinum nanoparticles

**Fig. 5:**
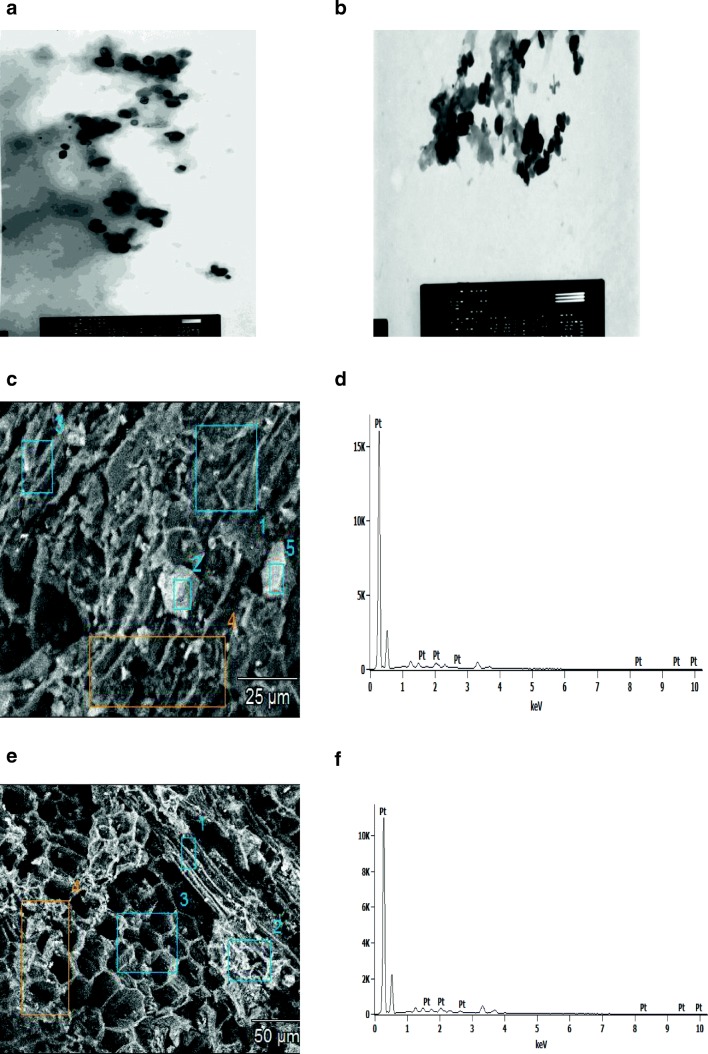
TEM and SEM images of synthesized platinum nanoparticles using *O.radix* extract at different scale bar (**a**) 200 nm (**b**) 500 nm, (**c**) 25 μm with (**d**) EDS profile and (**e**) 50 μm with (**f**) EDS profile

Transmission Electron Microscope Analysis (TEM) and Scanning Electron Microscopy (SEM) measurements were used to determine the size, shape and morphological features of platinum nanoparticles synthesized using *O.radix* extract. Transmission Electron Microscopy images (Fig. 5a and b) confirmed the presence of spherical and hexagonal platinum nanoparticles. Scanning Electron Microscopy (SEM) determined the presence of platinum nanoparticles whose size was about 20 nm (Fig. 5c and e). Şahin et al. (2018) who synthesized platinum nanoparticles with the use of pomegranate extract, also obtained spherical shape and a similar size. Pal et al. (2014) produced spherical platinum nanoparticles by means of microwave irradiation. Spherical nanoparticles were also obtained by Syed and Ahmad (2012), who conducted the biosynthesis of platinum nanoparticles using the fungus *Fusarium oxysporum.* Quasi-spherical shape of platinum nanoparticles was obtained by Kora and Rastogi (2018), who carried out the synthesis using gum olibanum *Boswellia serrata*. Sheny et al. (2013), using the leaves of *Anacardium occidentale*, synthesized crystalline and irregular rod-shaped Pt nanoparticles*.* Those results prove that there is a strong correlation between the shape of nanoparticles and the biological material used, i.e. the active substances in a given plant. According to Huang et al. (2007), when a larger concentration of bio-reducing agent is used, there occurs a relatively fast nucleation process, which is followed by a slower growing stage due to stronger interactions between protective bio-molecules and growth, leading to the formation of more isotropic particles.

The work also presents the EDS profile, which provides additional evidence of the reduction of platinum nanoparticles. Figure 5b and d depicts seven peaks between 0 kV and 10 kV. Those results indicated that the reaction product was composed of high purity platinum nanoparticles.

### Atomic force microscopy (AFM) studies of platinum nanoparticles

**Fig. 6:**
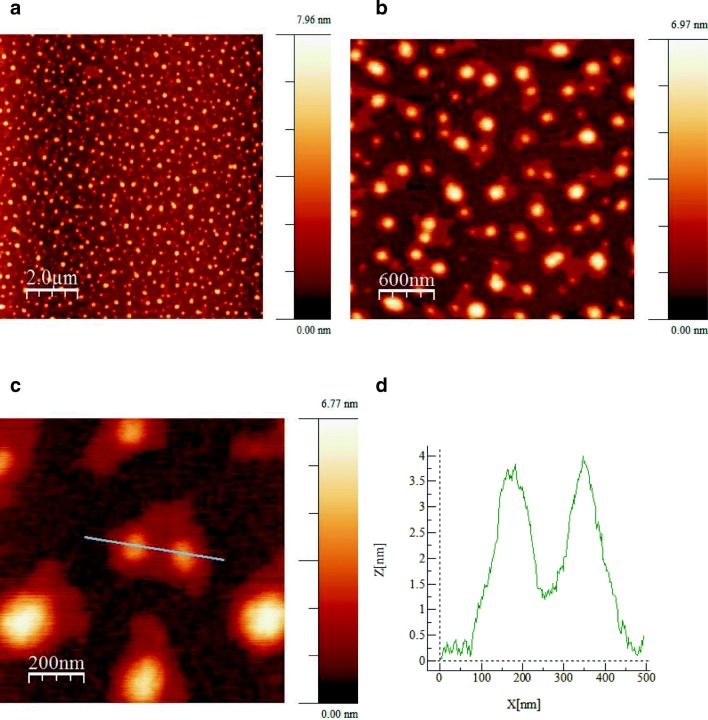
AFM images of synthesized platinum nanoparticles using *O.radix* extract (**a**) the topography for AFM 10 μm × 10 μm, **b** the topography for AFM 3 μm × 3 μm, **c** the topography for AFM 1 μm × 1 μm with the section by nanoparticles and (**d**) intersection for AFM 1 μm × 1 μm

The size and shape of the obtained platinum nanoparticles were confirmed by means of atomic force. The obtained results made it possible to determine the presence of platinum nanoparticles with the same shape as shown before by means of Transmission Electron Microscopy. However, Atomic Force Microscopy indicated the presence of smaller platinum nanoparticles, whose size was about 4 nm. Higher results obtained by means of TEM and SEM measurements stem from the agglomeration of nanoparticles. As we are dealing with biological material, particles may agglomerate. Figure 6 presents AFM image of synthesized platinum nanoparticles using *O. radix* with (A) the topography of 10 μm × 10 μm, (B) the topography of 3 μm × 3 μm, (C) the topography of 1 μm × 1 μm with the section by nanoparticles and (D) the profile for AFM 1 μm × 1 μm.

### Results of the cell cycle analysis

Mohammadi et al. showed that platinum nanoparticles have anticancer properties (Mohammadi et al. 2013). They examined the cytotoxicity of platinum nanoparticles by MTT assay on MCF-7 and HepG-2 cell lines. The obtained results demonstrated concentration-dependent toxicity for the tested cells. Moreover, platinum nanoparticles exhibited potent anticancer activity against PA-1 cell line via induction of apoptosis and cell cycle arrest (Bendale et al. 2017). According to the literature, the anticancer activity of platinum nanoparticles is associated with the inhibition of DNA replication and mitosis by the addition of platinum nanoparticles to the DNA strand (Sawosz et al. 2010). Platinum nanoparticles penetrate into the nucleus and mitochondria, binding with DNA molecule and leading to cellular apoptosis (Ullah et al. 2017).

**Fig. 7:**
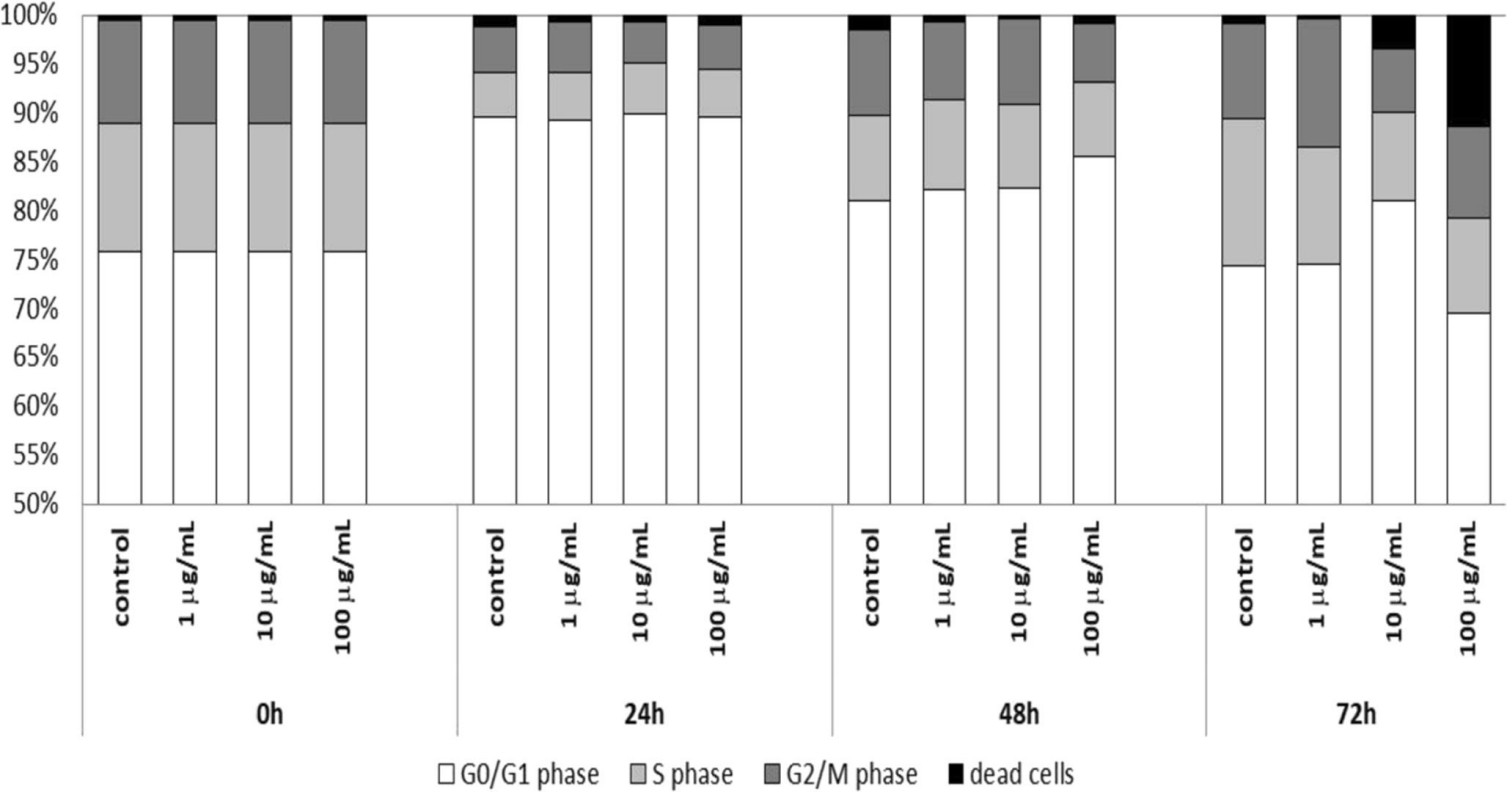
Analysis of the cell cycle of the A549 line cultured under the influence of synthesized platinum nanoparticles in following time points of 24 h, 48 h, and 72 h

**Fig. 8:**
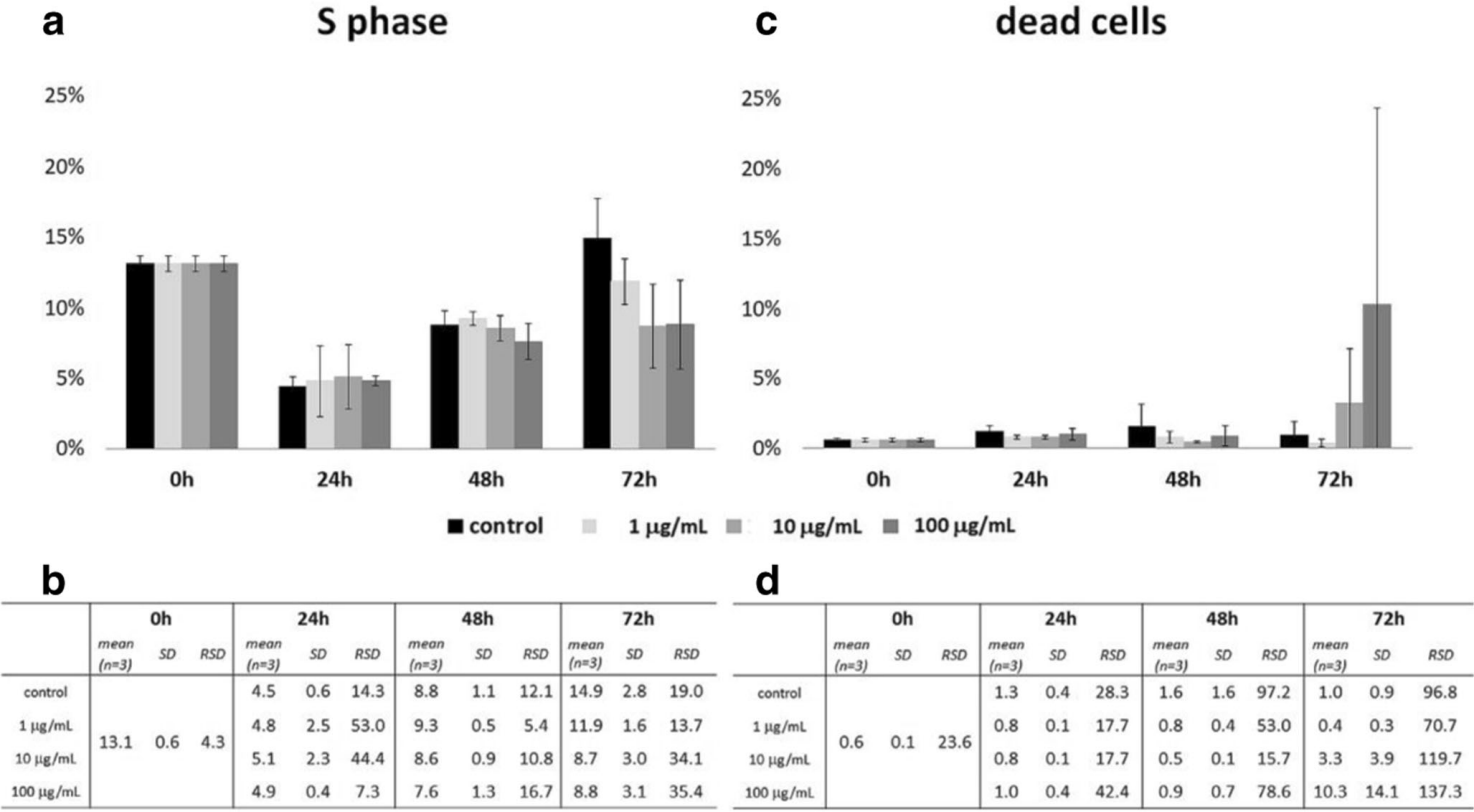
Evaluation of the percentage of A549 cells cultured under the influence of synthesized platinum nanoparticles in the S phase of the cell cycle and the proportion of dead cells

Al-Radadi and Najlaa (2019) demonstrated the anticancer activity of platinum nanoparticles against different cancer cells, including the colon carcinomab cells (HCT-116), breast cells (MCF-7), and hepatocellular carcinoma (HePG-2). In this study, the influence of platinum nanoparticles on human non-small cell lung carcinoma cells A549 was examined. Performed analysis of the cell cycle showed increasing of mortality of cells cultured together with the synthesized platinum nanoparticles. In the beginning, the proliferative activity of A549 cells under the influence of studied substance was higher as compared to the control. In the course of time of the culture, the percentage of dead cells was more significant (Fig. 7). The maximum mortality of cells was observed in the 72 h of the test in the sample where cells were cultured with the highest concentration of the platinum nanoparticles, 100 μg/mL. The grown percentage of dead cells was observed already in the 72 h under the influence of platinum nanoparticles molecules at the concentration of 10 μg/mL. Also in the 72 h of culture, the synthesized platinum nanoparticles in the concentration of 1 μg/mL inhibited the proliferation activity of studied cells, which observed value was lower as compared to the control cells. The proliferative activity of A549 cells decreased gradually over time in proportion to the increasing concentration of the test substance (Fig. 8). The bar graph of the percentage of cells in the S phase of the cell cycle compared to the percentage of dead cells clearly shows the relationship, where the inhibition of replication is associated with the increase the mortality increase. Observed phenomena are dependent on the time of cultivation and the concentration of the test substance. However, obtained results were not statistically important when they were tested with one-way non-parametric ANOVA (Kruskal-Wallis test).

## Conclusion

The biological methods for synthesizing metal nanoparticles have attracted more interest in recent years. This stems, among others, from the fact that the use of such methods may eliminate or reduce the amount of waste generated by chemical and physical methods. A great advantage of this method is the low cost of the metal ion reduction process. For this reason, in this work, the biological synthesis of platinum nanoparticles was conducted using *O. radix* extract. The techniques for measuring platinum nanoparticles synthesized using *O. radix* extract, such as AFM, TEM and SEM with EDS analyzer, confirmed the presence of nanoparticles with the size of 4 nm, which were locally agglomerated. The emergence of nanoscience has provided promising results in recent years by intersecting with various other branches of science and exercising impact on all forms of life. Nanosciene offers solutions to technological and environmental challenges in medicine. The conducted study showed the influence of platinum nanoparticles on human non-small cell lung carcinoma cells A549. The maximum mortality of cells was observed in the 72 h of the test, in the sample where cells were cultured with the highest concentration of platinum nanoparticles. Additionally, the proliferative activity of A549 cells decreased gradually over time in proportion to the increasing concentration of the platinum nanoparticles.
